# Behavioral, neuromorphological, and neurobiochemical effects induced by omega-3 fatty acids following basal forebrain cholinergic depletion in aged mice

**DOI:** 10.1186/s13195-020-00705-3

**Published:** 2020-11-16

**Authors:** Debora Cutuli, Eugenia Landolfo, Annalisa Nobili, Paola De Bartolo, Stefano Sacchetti, Doriana Chirico, Federica Marini, Luisa Pieroni, Maurizio Ronci, Marcello D’Amelio, Francesca Romana D’Amato, Stefano Farioli-Vecchioli, Laura Petrosini

**Affiliations:** 1 IRCCS Fondazione Santa Lucia, Rome, Italy; 2 University of Rome “Sapienza”, Rome, Italy; 3 University “Campus Bio-Medico”, Rome, Italy; 4 Department of Human Sciences, Guglielmo Marconi University, Rome, Italy; 5 Institute of Biochemistry and Cell Biology, CNR, Monterotondo, Italy; 6 Università Cattolica del Sacro Cuore, Rome, Italy; 7 IRCCS Fondazione Policlinico Universitario Agostino Gemelli, Rome, Italy; 8 Department of Pharmacy, University G. D’Annunzio of Chieti-Pescara, Chieti, Italy

**Keywords:** Aging, Cholinergic system, Omega-3 fatty acids, Memory deficits, Diet, Alzheimer’s disease

## Abstract

**Background:**

In recent years, mechanistic, epidemiologic, and interventional studies have indicated beneficial effects of omega-3 polyunsaturated fatty acids (n-3 PUFA) against brain aging and age-related cognitive decline, with the most consistent effects against Alzheimer’s disease (AD) confined especially in the early or prodromal stages of the pathology.

In the present study, we investigated the action of n-3 PUFA supplementation on behavioral performances and hippocampal neurogenesis, volume, and astrogliosis in aged mice subjected to a selective depletion of basal forebrain cholinergic neurons. Such a lesion represents a valuable model to mimic one of the most reliable hallmarks of early AD neuropathology.

**Methods:**

Aged mice first underwent mu-p75-saporin immunotoxin intraventricular lesions to obtain a massive cholinergic depletion and then were orally supplemented with n-3 PUFA or olive oil (as isocaloric control) for 8 weeks. Four weeks after the beginning of the dietary supplementation, anxiety levels as well as mnesic, social, and depressive-like behaviors were evaluated. Subsequently, hippocampal morphological and biochemical analyses and n-3 PUFA brain quantification were carried out.

**Results:**

The n-3 PUFA treatment regulated the anxiety alterations and reverted the novelty recognition memory impairment induced by the cholinergic depletion in aged mice. Moreover, n-3 PUFA preserved hippocampal volume, enhanced neurogenesis in the dentate gyrus, and reduced astrogliosis in the hippocampus. Brain levels of n-3 PUFA were positively related to mnesic abilities.

**Conclusions:**

The demonstration that n-3 PUFA are able to counteract behavioral deficits and hippocampal neurodegeneration in cholinergically depleted aged mice promotes their use as a low-cost, safe nutraceutical tool to improve life quality at old age, even in the presence of first stages of AD.

## Introduction

Omega-3 polyunsaturated fatty acids (n-3 PUFA) are essential dietary nutrients that constitute the major components of neuronal membranes and are key modulators of many neural functions throughout life [1–4]. Their daily intake could be mainly from plant-derived alpha-linolenic acid (ALA) and from fish- and marine-derived eicosapentaenoic acid (EPA) and docosahexaenoic acid (DHA), and their supplements [5, 6]. Unfortunately, nutritional research indicates that the “Western pattern diet” does not provide the brain with an optimal supply of n-3 PUFA, and aging per se is associated to a decrease in cerebral n-3 PUFA due to a reduced absorption, an inefficient biological ability to make long-chain n-3 PUFA (as EPA, DHA, and docosapentaenoic acid, DPA) from shorter chained fatty acids (as ALA), and a diminished n-3 PUFA capacity to cross the blood-brain barrier [7–9]. When diet does not provide enough n-3 PUFA, vulnerability to several diseases can increase [6]. Therefore, especially during aging, dietary interventions aimed to better balance these fatty acids could be important.

The ever-increasing number of elderly people translates into increasing demands for social-health and care services, particularly with respect to age-related neurodegenerative diseases, such as Alzheimer’s disease (AD). AD is the most common progressive dementia in people over the age of 65 years [10, 11]. Major symptoms of AD are memory loss, speech and language impairment, abstract reasoning decline, and mood changes [12, 13]. The main neuropathological hallmarks of AD include extracellular deposits of amyloid-β (Aβ), intracellular neurofibrillary tangles of tau protein, glial responses, and neuronal and synaptic loss in the limbic system, neocortical regions, and basal forebrain areas [12, 14, 15]. In AD patients, hippocampal-dependent functions are severely compromised and in vivo and post-mortem studies have reported remarkable shrinkage of the hippocampus [16–18]. Moreover, alterations in adult hippocampal neurogenesis have been reported at early AD stages, before the generalized presence of senile plaques or neurofibrillary tangles in the dentate gyrus (DG) [19–21].

The etiology of AD is still unknown, and no effective therapy has been yet identified to defeat the disease. Currently, there are only symptomatic treatments to attenuate AD-related cognitive deterioration which have no effects on AD progression [22–24], and vaccines are not yet available [25]. On this basis, there is substantial interest in identifying lifestyle factors, such as diet, capable of preventing or at least delaying cognitive decline at old age [26].

Current animal and human evidence, although somewhat inconsistent, indicates that n-3 PUFA supplementation may be beneficial against age-related dysfunctions. Specifically, in animal studies, learning and memory abilities as well as neurogenic and synaptogenic functions can be ameliorated by n-3 PUFA treatment during aging and in AD preclinical models [2, 3, 20, 27–30]. With regard to human studies, some observational and epidemiological studies and recent meta-analyses reported that n-3 PUFA intake is associated with improved cognition in older adults and in patients with mild cognitive impairment (MCI) [31–33], and with a lower risk of dementia and AD [27, 34–37]. Anyway, interventional studies showed contradictory results on the relationship between n-3 PUFA administration and cognitive performances during aging, with some studies succeeding [38–43] and other studies failing [38, 43–47] in revealing significant beneficial cognitive effects in older adults and patients with MCI and AD. Notably, comprehensive systematic reviews of the literature on randomized controlled trials reveal that there is no consistent evidence to support the effectiveness of n-3 PUFA supplementation in improving cognitive functions in AD patients, especially in case of advanced AD [36, 48–50]*.* When present, cognitive improvements associated with n-3 PUFA supplementation have been mostly demonstrated in patients with very mild AD [48, 51, 52]*.*

The intracerebroventricular (i.c.v.) injection of the mu-p75-saporin (saporin) immunotoxin is a valid animal model to partially mimic early AD pathology in mice, since the loss of integrity of the basal forebrain cholinergic system is one of the most reliable hallmarks of AD pathology [53]. Namely, the saporin immunotoxin provokes a selective and permanent removal of basal forebrain cholinergic inputs to the hippocampus, the entire cortical mantle, the amygdala, and the olfactory bulb [54–56].

Furthermore, animal studies offer better possibilities for controlled n-3 PUFA supplementation than interventional studies in humans allowing on one hand to better manage confounding factors, such as disease stage, age, cooking processes, other dietary components, socio-economic status, genetic background, healthy habits (e.g., exercise, not smoking, good sleep, social support, use of vitamin supplement, etc.), and on the other hand to better analyze the neural mechanisms underlying the eventual cognitive and behavioral improvements observed in the animal models.

Thus, in the present study, we used saporin immunotoxin i.c.v. injections as experimental model of AD first stages, which have been demonstrated to be the most crucial phase to observe n-3 PUFA beneficial effects against AD pathology in humans [51]. We then investigated the impact of an 8-week oral post-lesional administration of a mixture of EPA, DHA, and DPA on the cognitive and behavioral performances and hippocampal degeneration induced by immunotoxic forebrain cholinergic lesions during aging. To this aim, we compared emotional, mnesic, and social performances as well as hippocampal morphological and biochemical correlates of cholinergically depleted or sham-lesioned aged mice supplemented with n-3 PUFA with those which received olive oil (used as isocaloric control). In particular, after the behavioral evaluation, the neurodegeneration of hippocampal networks was analyzed by measuring neurogenesis levels in the DG and volumes and astrogliosis in the hippocampus, which is one of the main target areas of the lesioned cholinergic projections from medial septum/diagonal band. A quantification of n-3 PUFA brain levels was also performed.

## Materials and methods

### Animals

C57BL/6 male mice (*n* = 57) purchased from Envigo (S. Pietro al Natisone, Italy) were used. At their arrival, the animals were 8–9 months old and they were all ex-breeders. The animals were group-housed (3–4 mice/cage) with controlled temperature (22–23 °C) and humidity (60 ± 5%), under a 12:12 h light/dark cycle (lights on at 07:00 a.m.), with food and water freely available throughout the study.

Animals were randomly assigned to the following experimental groups:

- Sham-lesioned aged mice supplemented with olive oil (sham oil, *n* = 14);
- Sham-lesioned aged mice supplemented with n-3 PUFA (sham n-3 PUFA, *n* = 16);
- mu-p75-saporin-lesioned aged mice supplemented with olive oil (sap oil, *n* = 12);
- mu-p75-saporin-lesioned aged mice supplemented with n-3 PUFA (sap n-3 PUFA, *n* = 15).

All efforts were made to minimize animal suffering and reduce the number of mice used, in accordance with the European Union Directive of September 22, 2010 (2010/63/EU). All experiments were approved by the Italian Ministry of Health (Legislative Decree No 682/2016).

### Experimental procedures

**Fig. 1:**
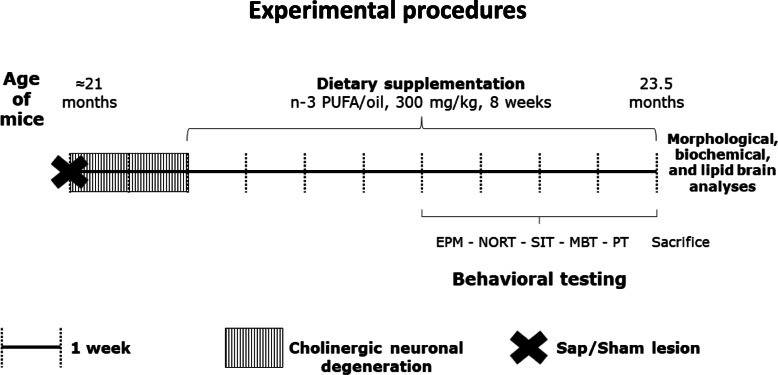
Experimental procedures. At ≈ 21 months of age, mice were subjected to i.c.v. injections of mu-p75-saporin or saline (sham lesion) to selectively deplete the forebrain cholinergic system. Two weeks after lesion, an 8-week oral supplementation (by gavage) with n-3 PUFA or olive oil began. After the first 4 weeks of dietary supplementation, the animals were behaviorally evaluated by means of a testing battery (elevated plus maze, EPM; novel object recognition task, NORT; social interactions test, SIT; marble burying test, MBT; Porsolt test, PT) lasting 4 weeks. At the end of testing, the mice were sacrificed, and brains collected for morphological, biochemical, and lipid analyses

To evaluate the potential therapeutic action of n-3 PUFA in the presence of a cholinergic depletion during aging, when ≈ 21 months old, mice were intraventricularly injected with the mu-p75-saporin immunotoxin (or saline) to induce (or not) a selective degeneration of basal forebrain cholinergic neurons. After 2 weeks, the animals began the supplementation by gavage with n-3 PUFA (or olive oil) lasting for 8 weeks. Four weeks after the gavage beginning, mice underwent the behavioral evaluation. At the end of gavage period, mice were sacrificed, and brains were collected for morphological, biochemical, and lipid analyses (Fig. 1).

### Surgery

The mu-p75-saporin is used to selectively deplete the central cholinergic system in mice. It is made by conjugating the low affinity p75 neurotrophin receptor (p75NTR) antibody to saporin, a ribosome inactivating protein. When the conjugate is internalized, mu-p75-saporin breaks away from the targeting agent and inactivates the ribosomes causing protein synthesis inhibition and, ultimately, cell death. In this way, the immunotoxin is able to eliminate cells expressing p75NTR in mouse as the cholinergic basal forebrain neurons, while sparing neighboring neurons that express glutamic acid decarboxylase, calbindin, and parvalbumin. The resulting permanent and selective saporin-dependent massive loss of cortical and hippocampal cholinergic afferents mimics neuropathological features and cognitive symptoms associated with MCI and early AD.

The mice assigned to the immunotoxic lesioned groups were intraventricularly injected with the mu-p75-saporin (Targeting Systems, San Diego, CA), while the sham-lesioned groups were intraventricularly injected with 0.9% saline.

Mice were anesthetized with a mixture of tiletamine/zolazepam (50 mg/kg Zoletil 100 i.p., Virbac s.r.l., Milan, Italy) and xylazine (10 mg/kg Rompun i.p., Bayer s.p.a., Milan, Italy). In the animals that had to be immunotoxic lesioned (*n* = 27), the mu-p75-saporin was injected through a 10-μl Hamilton syringe in each lateral ventricle (total dosage: 0.6 μg/mouse [54, 57], coordinates: antero-posterior (AP) = − 0.6 mm (from the bregma); medio-lateral (ML) = ±1 mm (from the midline); dorso-ventral (DV) = − 2.2 mm (from the dura) [58]. The immunotoxin (0.3 μl per side) was injected at a rate of 0.1 μl/min. At the end of injection, the needle was left in situ for 4 min to allow the diffusion.

In the remaining mice used as sham-lesioned controls (*n* = 30), 0.9% saline (0.3 μl per side) was injected into each lateral ventricle with the same injection procedure.

### Dietary manipulations

Two weeks after lesioning (time required to allow the immunotoxin to permanently deplete cholinergic neurons [54]), mice were supplemented by gavage for 8 weeks with a mixture of n-3 PUFA (containing 52% EPA, 39.2% DHA and 6% DPA; Meaquor 900, UGA Nutraceuticals, Italy) or olive oil (used as isocaloric control, containing 14.6% saturated fatty acids, 68.3% monounsaturated fatty acids and 8.7% PUFA of which 0.6% n-3 PUFA, i.e., ALA; De Cecco, Italy) at a 300 mg/kg dosage.

All animals were fed ad libitum with standard food pellets (Mucedola 4RF21 standard diet; Mucedola, Italy).

### Behavioral testing

Four weeks after n-3 PUFA (or olive oil) supplementation beginning (time expected to incorporate n-3 PUFA in the neuronal membranes [59, 60] and to habituate to gavage procedure), the animals underwent the following behavioral battery of validated tests tapping distinct cognitive and emotional functions: novel object recognition task (NORT) to evaluate recognition memory, elevated plus maze (EPM) and marble burying test (MBT) to measure anxious behaviors, social interactions test (SIT) to assess social behaviors, and Porsolt test (PT) to analyze depressive-like behaviors (Fig. 1). Half of each experimental group was exposed to NORT or MBT first alternatively, to control the effect of novelty—i.e., objects or marbles, while the other tests were administered in the same order as in Fig. 1.

All tests were performed between 10:00 a.m. and 06:00 p.m. All animals were subjected to handling habituation prior to the behavioral testing. Animals were tested in a pseudo-random order during each different task.

### Elevated plus maze (EPM)

The elevated plus maze (EPM) is a validated test to measure anxiety levels in rodents based on their natural proclivity toward dark, enclosed spaces and aversion for heights/open spaces [61–64]. In aging mice, anxiety is expected to increase [65]. Furthermore, cholinergic manipulations are known to modify anxiety levels [66, 67]. The maze consisted of a wooden cross-shaped structure elevated 60 cm above the floor, with a central platform (5 × 5 cm) and four 30 × 5 cm arms. The two oppositely positioned closed arms were enclosed by walls 20 cm high, while the two oppositely positioned open arms had no walls.

During a 5-min trial, the mouse was placed in the central platform and allowed to freely explore the apparatus. The maze was cleaned with a solution of 10% ethanol after each trial to remove olfactory clues. Trials were recorded by a ceiling-mounted camera and analyzed by a video analyzer (EthoVision XT, Noldus, The Netherlands). The following parameters were measured: total entries and total time spent in the open and closed arms; number of defecations.

### Novel object recognition task (NORT)

The novel object recognition task (NORT) is a validated test to measure recognition memory in rodents that exploits their natural tendency to explore novel items [68] and is strictly dependent on hippocampal integrity [69, 70].

The apparatus consisted of a chamber made of transparent Plexiglas (56 × 42 × 21 cm). The test was composed of three 5-min trials: habituation, training, and test trial [71, 72]. During habituation, mice were allowed to explore the empty chamber. Afterwards, mice were put in their home cages for 3 min. Then, during training trial, they were exposed to the now-familiar chamber containing two identical objects (objects A and B; two white Plexiglas 6.5-cm diameter spheres fixed to a 4 mm thick transparent squared base). One hour later, mice were once again exposed to the familiar chamber containing one familiar object (object A) and a novel object (a white cube of 5.5-cm side fixed to a 4 mm thick transparent squared base) to test long-term recognition memory (test trial). Contact time was considered to have occurred when the animal explored the object for at least 1 s. To balance for side bias, we randomly put each animal into the testing chamber from the opposite long sides of the apparatus (with the snout against the wall), so that if for one animal the object A was on the right and the novel object (and previously the object B) on the left, conversely for another animal inserted in the apparatus from the opposite side the object A was on the left and the novel object (and previously the object B) on the right.

The arena was cleaned with a solution of 10% ethanol after each trial to minimize olfactory signals.

A video camera connected to a monitor and to the image analyzer (EthoVision XT, Noldus, The Netherlands) was placed on top of the apparatus. To assess novel object recognition (novelty) memory the time spent exploring each object during the test trial was recorded and a discrimination index was calculated:

$$ \frac{\mathrm{contact}\ \mathrm{time}\ \mathrm{with}\ \mathrm{the}\ \mathrm{novel}\ \mathrm{object}\ (Tno)-\mathrm{contact}\ \mathrm{time}\ \mathrm{with}\ \mathrm{the}\ \mathrm{familiar}\ \mathrm{one}\ (Tfo)}{\mathrm{total}\ \mathrm{contact}\ \mathrm{time}\ \mathrm{with}\ \mathrm{object}\mathrm{s}\ \left( Tno+ Tfo\right)} $$

Further explorative parameters were the distance traveled in the arena (as index of horizontal exploration) and the number of rearings and wall-rearings (as index of vertical exploration). As emotional parameters, we considered grooming time and number of defecations.

### Marble burying test (MBT)

The marble burying test (MBT) is a validated test to measure the neophobic, anxious, and repetitive behaviors of mice [73, 74]. We used this test to evaluate the eventual effects on anxiety following the cholinergic lesion in aged mice [67]. The apparatus consisted of a rectangular cage made of transparent Plexiglas (40 × 24 × 17 cm) filled with sawdust 5 cm deep, without food and water. Nine glass marbles (1.5 cm in diameter) were equidistantly spaced on the flattened surface of the bedding in a 3 × 3 grid. During the test, each mouse was allowed to explore the apparatus for 30 min. At the end of the test, the number of successfully buried marbles was counted. A marble was considered buried when at least 2/3 of its size was covered with sawdust [74, 75].

### Social interactions test (SIT)

The social interactions test (SIT) is used to investigate social behaviors, which are known to decrease with age in rodents [65, 76]. Moreover, a selective cholinergic depletion of neocortex is reported to cause a significant decrease in the duration of active social interaction with an unfamiliar male in adult rats [77].

We isolated all males for 24 h in clean cages. The experimental trial started when an unfamiliar female mouse (2-month old and in the oestrus phase) was placed into the male’s cage for 10 min. A video camera was placed in front of the apparatus and videos were analyzed with the Observer software (Noldus, The Netherlands). Non-social, social, and sexual males’ behaviors were evaluated [78]. Non-social behaviors included exploration, rearing, wall-rearing, sniffing, digging, and self-grooming. Social behaviors included social investigation (following, sniffing head, body, and ano-genital region of the partner) and allogrooming (grooming the partner). Sexual behavior included mounts and pelvic thrusts.

### Porsolt test (PT)

The Porsolt test (PT) is a validated test evaluating depressive-like behaviors and coping strategies [79, 80]. In the present study, we assessed depressive-like behavior with PT since depression is frequently identified in elderly individuals, also at subthreshold levels and with a high prevalence in the community-dwelling elderly individuals [81, 82].

The apparatus consisted of a glass cylinder (diameter 18 cm, height 40 cm) containing 20 cm water at 28 ± 2 °C. The mice were submitted to a 5-min pre-test session. Twenty-four hours later, mice were tested for the second time in the same apparatus for 5 min (test session) [83]. At the end of each session, mice were removed from the cylinder, placed under a heat source for a while and allowed to dry in small cages. Then, the mice were brought back to their home cages. Animals’ behavior was recorded by using a frontally mounted video camera. Later, an observer blind to the specimen’s group manually scored the videos (EthoVision XT, Noldus, The Netherlands).

The total immobility time was recorded as index of behavioral despair. Immobility was defined as the total absence of any movements except for those necessary to float and for respiration.

### Morphological analyses

#### Bromodeoxyuridine (BrdU) treatment and immunocytochemistry

In aging, the DG of the hippocampus exhibits a severe decrease of progenitor proliferation, resulting in progressive decline of adult neurogenesis [84, 85]. The neuroinflammatory response after injury is characterized by a striking increase of glial fibrillary acidic protein positive (GFAP^+^) astrocytic population, a process named astrocytosis [86, 87]. In the present study, we assessed n-3 PUFA effects on the proliferation and differentiation of newborn neurons as well as on the number of GFAP^+^ cells in the DG of the four experimental groups.

Five mice per group received daily i.p. injections of 50 mg/kg of BrdU dissolved in saline (0.9% NaCl adjusted to pH 7.2 with NaOH) for 5 days. One day after the final injection, the animals were sacrificed and transcardially perfused, under deep anesthesia with a mixture of tiletamine/zolazepam (500 mg/kg Zoletil 100 i.p., Virbac s.r.l., Milan, Italy) and xylazine (100 mg/kg Rompun i.p., Bayer s.p.a., Milan, Italy) with 4% paraformaldehyde (PFA) in 0.1 M phosphate buffer (PBS). The brains were removed and kept overnight in 4% PFA. Afterwards, brains were equilibrated in 30% sucrose and cryopreserved at − 80 °C.

Brains embedded in Tissue-Tek OCT (Sakura, USA) were cut by cryostat at − 25 °C in 40 μm coronal serial free-floating sections. To detect BrdU, DNA was denatured with 2 N HCl for 40 min at 37 °C to facilitate antibody access, followed by 0.1 M borate buffer (pH 8.5) for 20 min. Sections were incubated overnight at 4 °C with a primary antibody rat anti-BrdU (AbD Serotec Cat# MCA2060) diluted 1:300 in Tris-buffered saline containing 0.1% Triton, 0.1% Tween 20, and 3% normal donkey serum (blocking solution).

For immunofluorescence analysis, the sections were then stained for multiple labeling by using fluorescent methods. After permeabilization with 0.3% Triton X-100 in PBS, the sections were incubated with 3% normal donkey serum in PBS for 16–18 h with the following primary antibodies: 1:200 goat polyclonal antibodies against doublecortin (DCX) (Santa Cruz Biotechnology, Inc. Cat# sc-8066), 1:300 goat polyclonal antibodies against sex-determining region Y-box 2 (SOX2) (Santa Cruz Biotechnology, Inc. Cat# 17320), and 1:500 mouse monoclonal antibodies against GFAP (SIGMA Cat# G6171). The secondary antibodies used to visualize the antigen were as follows: 1:200 donkey anti-rat Cy3-conjugated (Jackson ImmunoResearch; BrdU), 1:200 donkey anti-goat Cy3-conjugated (Jackson ImmunoResearch DCX, SOX2), and 1:200 donkey anti-mouse Cy2-conjugated (Jackson ImmunoResearch GFAP).

Images of the immunostained sections were obtained by laser scanning confocal microscopy using a TCS SP5 microscope (Leica Microsystem, Germany).

Analyses were performed in sequential scanning mode to rule out cross-bleeding between channels.

#### Quantification of cell number

To estimate the number of positive cells in the DG, we combined confocal z-stack imaging and optical dissector analysis as established by others [88, 89].

Slices were collected using systematic random sampling. The brain was coronally sliced in rostro-caudal direction, thus including the dorsal hippocampal region (approximately from bregma − 1.06 to − 2.54 [90]). Approximately total of 40 coronal sections with 40 μm of thickness were obtained from each brain; about 1-in-6 series of sections (each slice thus spaced 240 μm apart from the next) were analyzed by confocal microscopy and used to count the number of cells expressing the different markers throughout the rostro-caudal extent of the dorsal hippocampus. Quantification of BrdU, SOX2, DCX, and GFAP^+^ cells in the dorsal DG has been obtained in every sixth coronal section (6 sections per brain) spanning the dorsal region of hippocampus. One optical section stack was obtained from three different sub-regions of the DG (the crest, the suprapyramidal, and the infrapyramidal blade) using a CLSM (TCS-SP2; Leica Microsystem) equipped with an oil immersion objective lens (× 63, NA 1.32). To allow an efficient optical dissector analysis, we optimized the sampling conditions as follows: pixel size = 0.30 × 0.30 μm, slice interval = 1.5 μm, slice number = 20, and unbiased counting frame size = 225 × 200 mm. These optical sections were projected, and a montage was composed of the projected sections with NIH ImageJ. Quantitative analysis of hippocampal cell populations was performed by means of design-based (assumption-free, unbiased) stereological method, the optical dissector [91]. The unbiased counting frame [92] was superimposed by using the Blend Images Plugin of ImageJ. With the lost cap bias taken into consideration, the start and end points of the optical dissector height were set at approximately 2 mm inside the cut surface of mounted tissue. Then, the optical section at the start point was used as a look-up section, and the remaining optical sections were used as reference sections. According to the optical dissector principle, all cells cut through the lookup section or touching an exclusion line were disregarded, and they were marked in one color using the Cell Counter plugin of ImageJ (NIMH). The cells cut exclusively through the reference sections were counted and marked in a different color, as long as they were touching an inclusion line or were completely within the frame.

The total number of markers within the single section of DG was obtained as:

$$ \mathrm{N}=\sum {\mathrm{Q}}^{-}/\left(\mathrm{h}/\mathrm{SV}\right) $$

where “∑Q^-^” was the number of dissector-counted cell, “*h*” was the height of the optical dissector, and “SV” was the volumetric shrinkage factor (0.65).

The estimation of the total DG cell numbers was obtained by multiplying the average number of positive cells per section by the total number of 40 μm sections [93–95].

#### Volumetric measurement

Rostro-caudal sections of the dorsal horn of hippocampus (approximately from bregma − 1.06 to − 2.54 [58]) of each animal were mounted onto glass slide and stained with 4′,6-diamidino-2-phenylindole (DAPI) for 1 min. Stained sections were viewed at low magnification by using Olympus BX53 digital photomicroscope. Digital images were then captured electronically and displayed on a computer screen. The volume of the total hippocampus and its subregions were unbiasedly estimated by means of point counting methods, using the Cavalieri’s principle [96, 97]. Briefly, a 200-μm^2^ point counting grid was superimposed on the images. The points hitting on the total hippocampus and its subfields (DG, CA1+CA3) were separately counted. The total volume of the hippocampus and its subfields was determined by applying the following formula:

$$ \mathrm{Volume}=\sum \mathrm{P}\ \mathrm{x}\ \mathrm{d}\ \mathrm{x}\ \mathrm{t}\ \mathrm{x}\ \mathrm{a}/\mathrm{p} $$

where “∑P” represented the number of points hitting the region analyzed, “*d*” was equal to the distance from one section to the next (*d* = 0.24 mm), “*t*” was the mean section thickness (*t* = 0.04 mm), and “a/p” was equal to the area associated with one point in the grid.

### Biochemical analyses

#### Total protein extraction

Hippocampi were isolated from the entire brain and tissues were homogenized in lysis buffer containing (in mM) 320 sucrose, 50 NaCl, 50 Tris-HCl pH 7.5, 1% Triton X-100, 1 sodium orthovanadate, 5 β-glycerophosphate, 5 NaF, and protease inhibitor cocktail. Homogenates were incubated on ice (30 min) and centrifuged at 15,000*g* at 4 °C (10 min) [98]. The total protein content of the supernatant was determined by the Bradford method.

#### Immunoblotting analysis

Proteins were applied to SDS-PAGE and electroblotted on a polyvinylidene difluoride membrane. Immunoblotting analysis was performed using a chemiluminescence detection kit. The relative levels of immunoreactivity were determined by densitometry using the ImageJ software.

The primary antibodies were as follows: vesicular acetylcholine transporter (VAChT) (1:500, Synaptic Systems, #139103), GFAP (1:1000, Dako, #Z0334), and Actin (1:10000, Millipore, #MABT825). The secondary antibodies were as follows: goat anti-mouse IgG (1:3000; Bio-Rad, # 1706516) and goat anti-rabbit IgG (1:3000; Bio-Rad, # 1706515).

Membranes were stripped using Re-Blot Plus Strong Solution (Millipore) for 15 min at room temperature.

### Lipid analysis

#### Analysis of n-3 PUFA by gas chromatography/mass spectrometry (GC/MS)

Fatty acids were extracted using the method reported by Folch [99] with slight modifications. Briefly, brains were homogenized in CHCl3/MeOH (2:1 v/v) to a final dilution of 20-fold of the original sample volume, assuming that the tissue has the same specific gravity of water. Five hundred nanograms per microliter of internal standard mixture composed by pentadecanoic acid, nonadecanoic acid, and eneicosanoic acid was added in all samples. The resulting organic phase was evaporated to dryness in a speed-vac at room temperature and then derivatized by using 25 μL 1% pentafluorobenzyl bromide in acetonitrile and 25 μL 1% diisopropylethylamine in acetonitrile and incubated for 20 min at room temperature [100].

Derivatized samples were dried down under vacuum by using speed vac and then transferred in the injection vials dissolved by 50 μL iso-octane.

GC/MS analyses were performed using Trace GC 1310 (Thermo Scientific, USA) equipped with 30 m × 0.25 mm fused silica capillary column SLB TM-5 MS (Supelco) and connected to ISQ Mass Spectrometer (Thermo Scientific, USA). Two microliters of each sample was injected in split mode (1:25), the injection temperature was set at 250 °C; the carrier gas was Helium (ultra-high purity) and the flow rate was maintained constant at 1 mL/min. The initial oven temperature was 150 °C, rising 10 °C/min to 270 °C, and then increased by 40 °C/min up to 310 °C, held for 1 min. Both mass transfer line and the ion source were kept constant at 280 °C. MS experiments were led by negative chemical ionization mode (NCI), using Methane (ultrahigh purity) as reagent gas with a constant flow of 0.5 mL/min and the quadrupole temperature was set at 150 °C. The analysis was performed by selected ion monitoring (SIM) mode low resolution, and the free fatty acids were identified in comparison with commercial standards.

All identification and quantification results were obtained by Xcalibur 3.1 software (Thermo Scientific, USA).

### Statistical analyses

All data were tested for normality (Shapiro-Wilk test). When normally distributed, data were analyzed by using three-way ANOVAs (with lesion and diet as between-factors and trial as within-factor) followed by Tukey post-hoc tests. When data were not normally distributed, non-parametric analyses (Kruskal-Wallis ANOVA, Wilcoxon test, Mann-Whitney *U* test) were used. Morphological data were analyzed by using Student’s *T* test. Values of *p* < 0.05 were considered significant (Statistica 12, Statsoft). The sample size was calculated based on a priori power analysis (1-β = 0.80, *α* error = 0.05, two tails; GPower 3.1). For example, to obtain a significant difference between two independent groups in the NORT discrimination index, or in the GFAP levels, or in the hippocampal volume, we should have used samples of at least 10, 2, or 3 animals, respectively. Considering an eventual increased mortality rate due to the age of the animals and the surgery, we used a greater number of animals in each group.

## Results

### Behavioral testing

### EPM

**Fig. 2:**
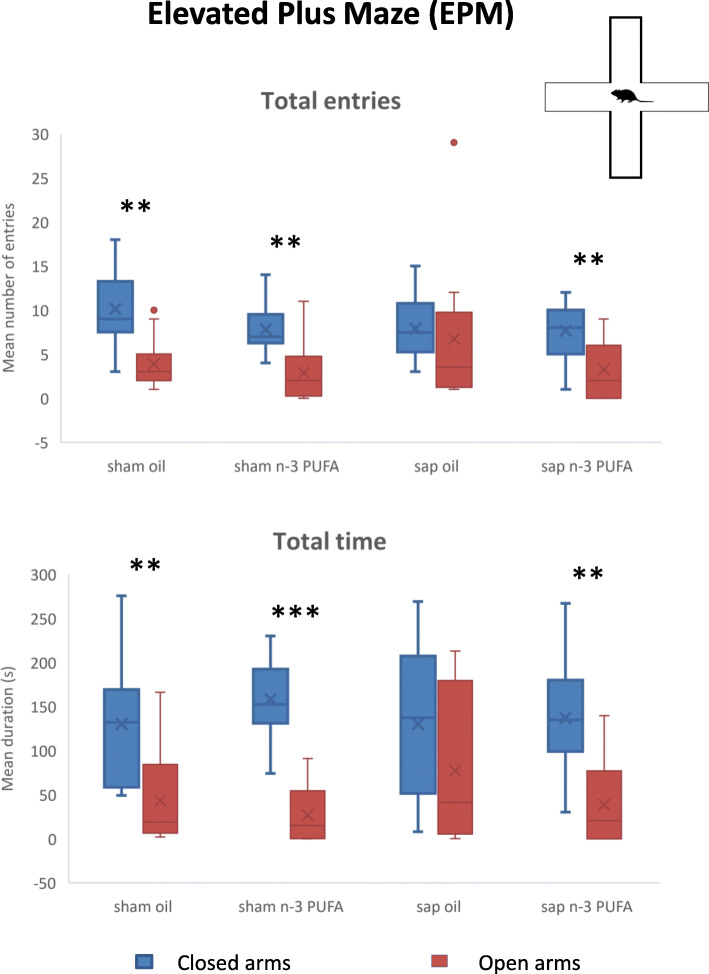
Elevated plus maze (EPM). Total entries and time spent in the closed and open arms by the four experimental groups (sham oil, *n* = 14; sham n-3 PUFA, *n* = 16; sap oil, *n* = 12; sap n-3 PUFA, *n* = 15). **p ≤ 0.01, ***p < 0.001. In this and the other figures, in box-and-whisker plots, the center line shows the median value, edges are upper and lower quartiles, whiskers show minimum and maximum values, crosses indicate mean values, and any external point is considered an aberrant value

Wilcoxon tests revealed that mice belonging to both sham-lesioned groups significantly entered more frequently (sham oil: *T* = 6.5, *p* = 0.004; sham n-3 PUFA: T = 8.5, *p* = 0.002) and spent more time (sham oil: *T* = 13, *p* = 0.01; sham n-3 PUFA: *T* = 1, *p* = 0.0005) in the closed vs. open arms. Conversely, sap oil mice showed no significant differences in total entries (sap oil: *T* = 28, *p* = 0.39) and time spent (*T* = 24, *p* = 0.24) in the closed vs. open arms (Fig. 2). Interestingly, the n-3 PUFA treatment was able to restore the expected aversion for the open arms in sap-lesioned mice (frequency: *T* = 14, *p* = 0.009; time: *T* = 6, *p* = 0.002). Number of defecations (mean and S.E.; sham oil = 1.43 ± 0.29; sham n-3 PUFA = 1.69 ± 0.42; sap oil = 1.42 ± 0.36; sap n-3 PUFA = 1.13 ± 0.19) did not differ among groups (Kruskal-Wallis ANOVA: *H* = 0.75, *p* = 0.86).

These results indicate that in aged mice treated with olive oil, the mu-p75-saporin lesion induced anxiolytic effects, while in the lesioned aged animals, the n-3 PUFA treatment was able to restore the expected aversion for the open arms.

### NORT

**Fig. 3:**
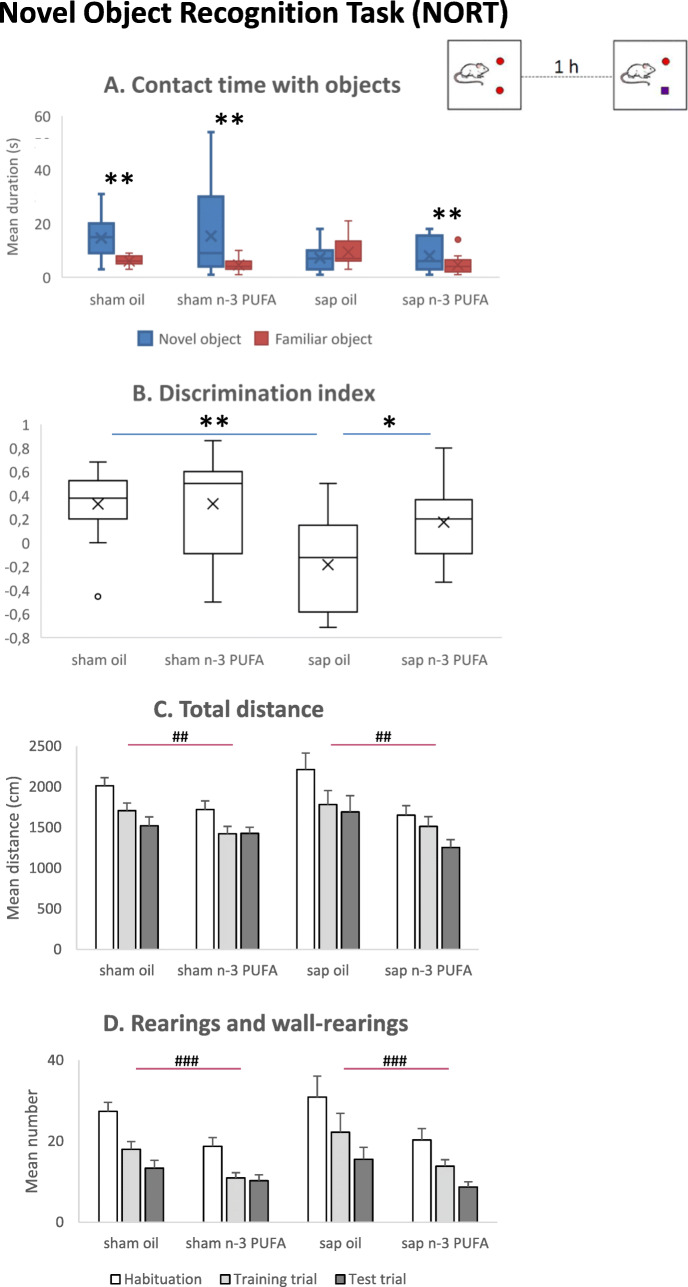
Novel object recognition task (NORT). Contact time with the novel and familiar objects (**a**), discrimination index (**b**), total distance (**c**), and total number of rearings (**d**) displayed by the four experimental groups (sham oil, *n* = 11; sham n-3 PUFA, *n* = 15; sap oil, *n* = 9; sap n-3 PUFA, *n* = 13). Asterisks indicate the level of statistical significance of the comparisons between contact time (s) with the novel vs. familiar object (**a**) and discrimination index between groups (**b**): **p ≤ 0.01, ***p < 0.001. Hashes indicate the statistical significance of diet effect (**c**, **d**): ##*p* < 0.01, ###*p* < 0.001. Post-hoc comparisons between trials in **c** (habituation vs. training or test trials, *p* < 0.001; training trial vs. test trial, *p* < 0.05) and **d** (habituation vs. training or test trials, *p* < 0.001; training trial vs. test trial, *p* < 0.01)

During the training trial, all mice equally explored (for about the 50% of total object contact time) the two identical objects (Kruskal-Wallis ANOVA, *H* = 0.03, *p* = 0.85; Suppl. Table 1). During the test trial, sham oil, sham n-3 PUFA, and sap n-3 PUFA mice showed a more evident preference for the novel object (around 64% of total object contact time) in comparison to sap oil mice (around 41% of total object contact time; Kruskal-Wallis ANOVA, *H* = 9.75, *p* = 0.02; Suppl. Table 1). In fact, Wilcoxon tests revealed a significant difference between the time spent in contact with novel vs. familiar object in all groups (sham oil: T = 2, *p* = 0.009; sham n-3 PUFA: T = 10, *p* = 0.004; sap n-3 PUFA: T = 10, *p* = 0.01), except for sap oil group (T = 10.5, *p* = 0.29; Fig. 3a). Moreover, Kruskal-Wallis ANOVA on the discrimination index was significant (*H* = 9.75, *p* = 0.02) and Mann-Whitney *U* comparisons between groups indicated that n-3 PUFA treated lesioned mice recognized object novelty as both sham control groups (sap n-3 PUFA vs. sham oil: *U* = 48, *p* = 0.17; sap n-3 PUFA vs. sham n-3 PUFA: *U* = 65, *p* = 0.13), while sap oil showed the worst recognition memory (sap oil vs. sham oil: *U* = 16.5, *p* = 0.01; sap oil vs. sap n-3 PUFA: *U* = 28.5, *p* = 0.04) (Fig. 3b).

As for explorative parameters, three-way ANOVAs (lesion x diet x trial) revealed significant effects of diet and trial on the horizontal exploration (total distance, lesion effect: *F*_1,44_ = 0.20, *p* = 0.65; diet effect: *F*_1,44_ = 8.92, *p* = 0.005; trial effect: *F*_2,88_ = 43.21, *p* < 0.0000001; lesion x diet: *F*_1,44_ = 0.85, *p* = 0.36; trial x lesion: *F*_2,88_ = 0.44, *p* = 0.64; trial x diet: *F*_2,88_ = 1.86, *p* = 0.16; trial x lesion x diet: *F*_2,88_ = 2.07, *p* = 0.13; Fig. 3c) and the vertical exploration (rearings and wall-rearings, lesion effect: *F*_1,44_ = 1.11, *p* = 0.30; diet effect: *F*_1,44_ = 13.40, *p* = 0.0007; trial effect: *F*_2,88_ = 56.30, *p* < 0.0000001; lesion x diet: *F*_1,44_ = 0.33, *p* = 0.60; trial x lesion: *F*_2,88_ = 0.99, *p* = 0.37; trial x diet: *F*_2,88_ = 1.93, *p* = 0.15; trial x lesion x diet: *F*_2,88_ = 0.14, *p* = 0.86; Fig. 3d).

As for emotional parameters, no differences were found among groups. In fact, both Kruskal-Wallis ANOVAs on grooming time (habituation: *H* = 2.11, *p* = 0.55; training trial: *H* = 0.72, *p* = 0.87; test trial: *H* = 1.91, *p* = 0.59) and number of defecations (habituation: *H* = 2.87, *p* = 0.41; training trial: *H* = 1.20, *p* = 0.75; test trial: *H* = 7.37, *p* = 0.06) failed to reveal any significant difference in any trial of the test (Suppl. Table 1).

These findings indicate that n-3 PUFA treatment counteracted the impairments in novelty recognition memory induced by the cholinergic depletion and reduced the horizontal and vertical exploration in both sham and immunotoxic lesioned aged mice.

### MBT

**Fig. 4:**
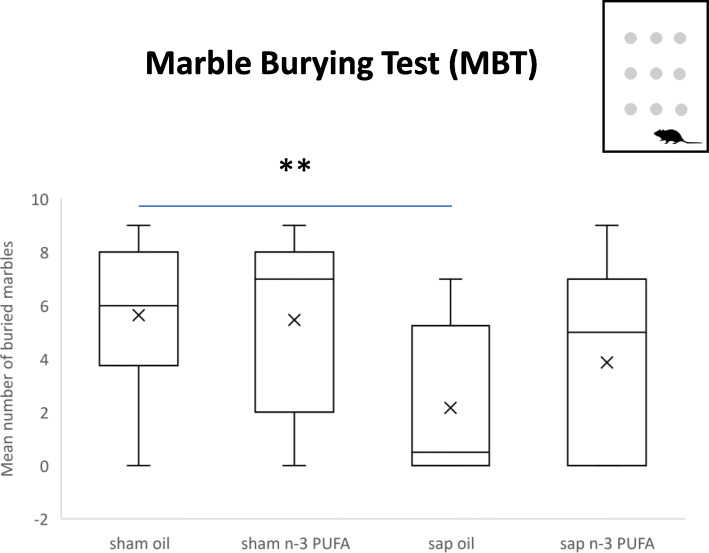
Marble burying test (MBT). Total number of marbles buried by the animals belonging to the four experimental groups (sham oil, n = 14; sham n-3 PUFA, n = 15; sap oil, n = 12; sap n-3 PUFA, n = 15). ** *p* = 0.01

Kruskal-Wallis ANOVA on the total number of marbles buried by the animals belonging to the four experimental groups was significant (*H* = 14.23, *p* = 0.03). Mann-Whitney *U* comparisons between groups indicated that both sham-lesioned groups buried the same number of marbles (*U* = 100, *p* = 0.82). Sap oil mice buried a lower number of marbles in comparison to sham oil (*U* = 36.5, *p* = 0.01). Sap n-3 PUFA mice did not differ from the remaining experimental groups (vs. sham oil: *U* = 80.5, *p* = 0.28; vs. sham n-3 PUFA: *U* = 75.5, *p* = 0.12; vs. sap oil: *U* = 70.5, *p* = 0.33) (Fig. 4).

These findings show that in aged mice the mu-p75-saporin lesion produced a reduced burying behavior (anxiolytic effect) that the n-3 PUFA treatment only partially rescued.

### SIT

Due to technical troubles during SIT data video recording, we were able to analyze the performance of a subset of animals only (sham oil, *n* = 9; sham n-3 PUFA, *n* = 9; sap oil, *n* = 7; sap n-3 PUFA, *n* = 10). Sexual behaviors and allogrooming were not observed in any group of mice. No differences in non-social behaviors [sum (mean and S.E.): sham oil = 302.07 ± 5.51 s; sham n-3 PUFA = 298.80 ± 8.51 s; sap oil = 283.28 ± 17.64 s; sap n-3 PUFA = 295.32 ± 8.07 s] and social behaviors [sum (mean and S.E.): sham oil = 29.98 ± 10 s; sham n-3 PUFA = 23.12 ± 7.74 s; sap oil = 30.92 ± 19.36 s; sap n-3 PUFA = 25.68 ± 6.47 s] were found among groups (Table 1).

**Table 1:** Social interactions test (SIT). Results of the non-parametric analyses (Kruskal-Wallis ANOVA) on duration (s) of non-social and social behaviors observed in the four experimental groups during SIT

**Non-social behaviors**	
Exploration	*H* = 4.70, *p* = 0.19
Rearing and wall-rearing	*H* = 7.16, *p* = 0.07
Sniffing	*H* = 1.48, *p* = 0.69
Digging	*H* = 2.30, *p* = 0.51
Self-grooming	*H* = 5.21, *p* = 0.16
Sum of non-social behaviors	*H* = 1.36, *p* = 0.71
**Social behaviors**	
Following	*H* = 1.15, *p* = 0.76
Head sniffing	*H* = 0.79, *p* = 0.85
Body sniffing	*H* = 4.45, *p* = 0.22
Ano-genital sniffing	*H* = 1.16, *p* = 0.76
Sum of social behaviors	*H* = 2.38, *p* = 0.50

Thus, as expected, the overall social interaction time of the experimental aged subjects with females was low, but not affected by cholinergic depletion or n-3 PUFA treatment.

### PT

**Fig. 5:**
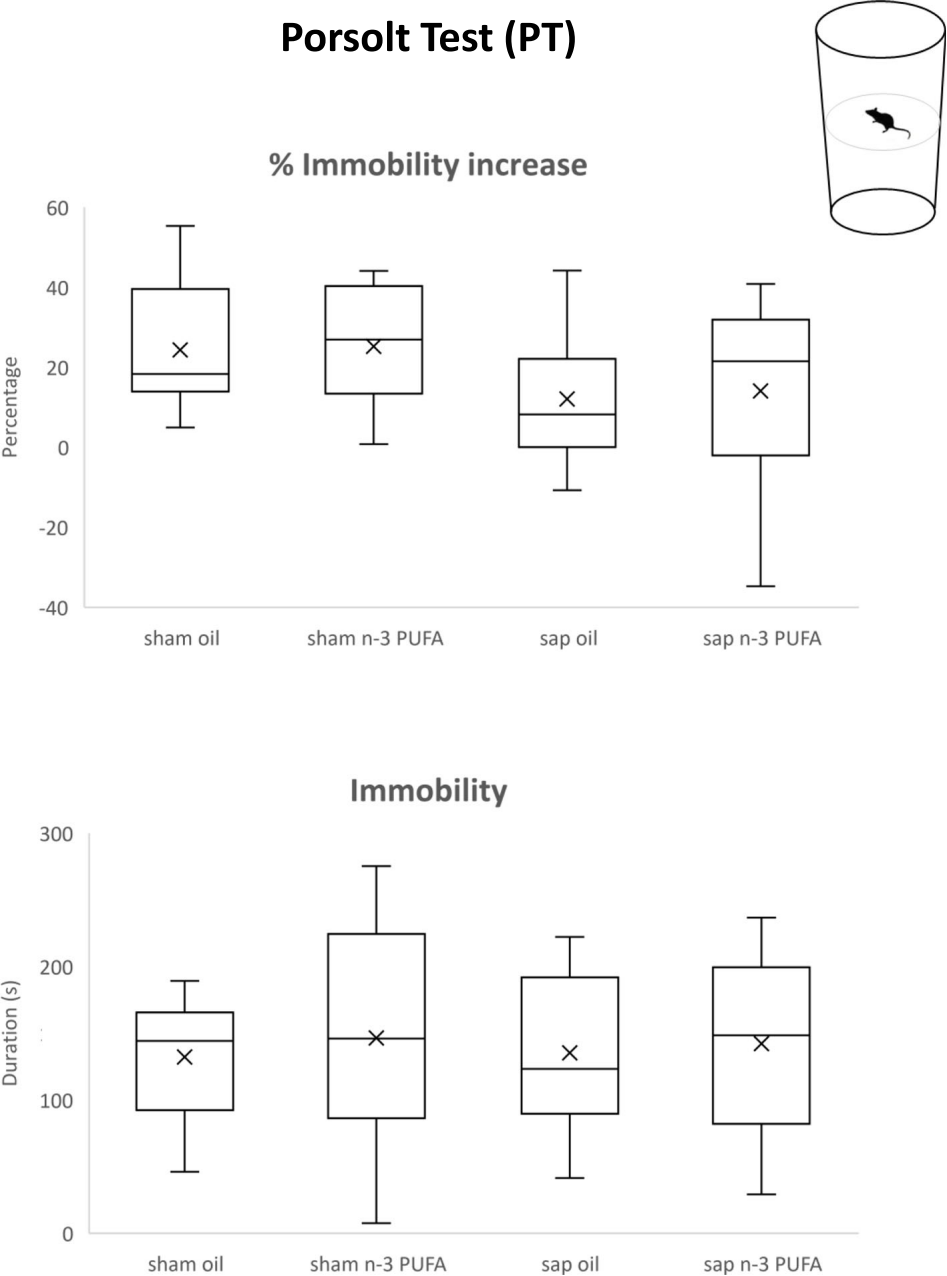
Porsolt test (PT). Percentual increase in immobility displayed between test session and pre-test session and duration (s) of immobility during the test session displayed by the four experimental groups (sham oil, *n* = 10; sham n-3 PUFA, *n* = 14; sap oil, *n* = 11; sap n-3 PUFA, *n* = 12)

As shown in Fig. 5, immobility time similarly increased in all groups between sessions (*H* = 5.17, *p* = 0.16) and no differences among groups were found in the duration of immobility during the test session (*H* = 0.33, *p* = 0.95). These findings indicate that in aged mice the re-exposure to an aversive condition induced a despair response affected by neither the cholinergic depletion nor the n-3 PUFA treatment.

### Morphological analyses

Considering the fact that in mice adult neurogenesis is predominant in the dorsal hippocampus respect to the ventral hippocampus [101–104] and that the neurodegenerative AD-like syndrome affects at a first stage the dorsal hippocampus and the dorsal hippocampus-dependent spatial learning and memory functions [105–107], we decided to analyze volume, neurogenesis, and astrogliosis parameters in the dorsal horn of the hippocampus.

### Hippocampal volume

**Fig. 6:**
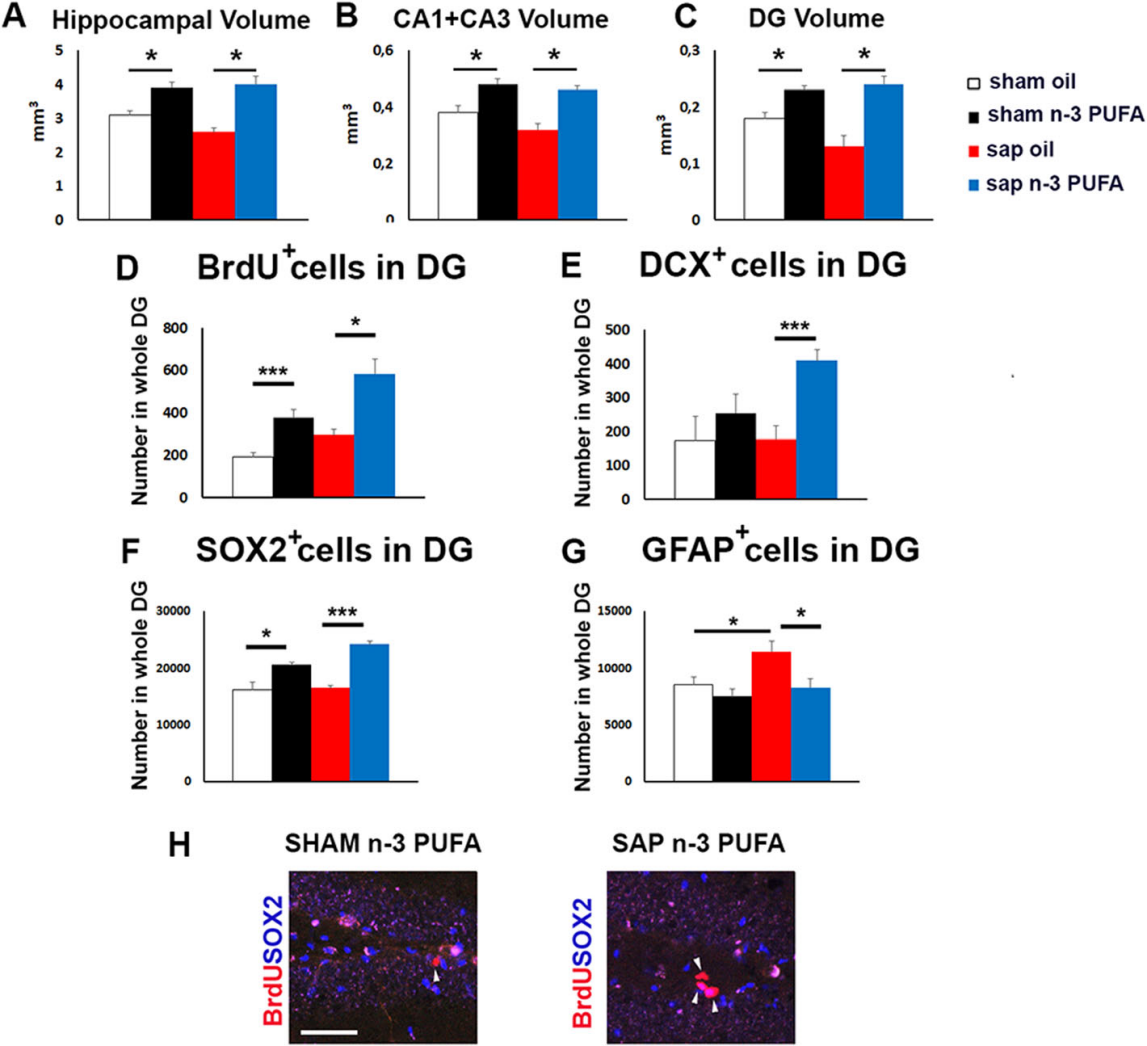
Morphological analyses. **a**–**c** Graphs showing the variations of volume in the whole hippocampus, CA1+CA3, and dentate gyrus (DG) in the four experimental groups. **d** Graph showing the large increase of BrdU^+^ cells in n-3 PUFA (sham and sap) groups when compared to their relative oil groups. **e** Graph representing the increased number of SOX2^+^ sub-population in the n-3 PUFA (sham and sap) groups in respect to their relative oil groups. **f** Graph showing the enhancement of DCX^+^ neuroblasts in sap n-3 PUFA group in comparison with sap oil group. **g** Graph indicating the increased number of GFAP^+^ cells in sap oil vs. sham oil mice and the decreased number of GFAP^+^ cells in sap n-3 PUFA vs. sap oil mice. **h** Representative images showing the increase of the BrdU^+^ cells (indicated by arrowheads) in the DG of the sham n-3 PUFA and sap n-3 PUFA groups. Scale bar 100 μm. *p < 0.05, ***p < 0.001. DG, dentate gyrus

In the dorsal region of the hippocampus, n-3 PUFA supplementation induced a significant volume preserving in both sham (mean and S.E.; sham oil = 3.12 ± 0.15 mm^3^; sham n-3 PUFA = 3.96 ± 0.32 mm^3^; sham n-3 PUFA vs. sham oil, *p* = 0.03; Fig. 6a) and mu-p75-saporin lesioned groups (sap oil = 2.75 ± 0.27 mm^3^; sap n-3 PUFA = 4.1 ± 0.43 mm^3^; sap n-3 PUFA vs. sap oil, *p* = 0.02; Fig. 6a). A similar volume increase has been observed in the CA1+CA3 regions (sham oil = 0.36 ± 0.02 mm^3^; sham n-3 PUFA = 0.48 ± 0.02 mm^3^; sap oil = 0.32 ± 0.02 mm^3^; sap n-3 PUFA = 0.47 ± 0.01 mm^3^; sham n-3 PUFA vs. sham oil, *p* = 0.02; sap n-3 PUFA vs. sap oil *p* = 0.04; Fig. 6b) and DG (sham oil = 0.17 ± 0.02 mm^3^; sham n-3 PUFA = 0.24 ± 0.01 mm^3^; sap oil = 0.14 ± 0.04 mm^3^; sap n-3 PUFA = 0.25 ± 0.03 mm^3^; sham n-3 PUFA vs. sham oil, *p* = 0.04; sap n-3 PUFA vs. sap oil, *p* = 0.04; Fig. 6c). No lesion effects were found on hippocampal volumes.

### Hippocampal neurogenesis

#### Neural stem cells (NSCs) and proliferation

To analyze the effects of n-3 PUFA treatment on dorsal hippocampal neurogenesis, the proliferation and differentiation of newborn neurons were investigated in the four experimental groups. As for the proliferation analysis, the number of BrdU^+^ proliferating progenitors in the DG was calculated. Saporin injection induced an evident although not significant increase in proliferation (mean and S.E.; sham oil = 193.1 ± 18.4 BrdU^+^ cells; sap oil = 294 ± 29.8 BrdU^+^ cells; sham oil vs. sap oil, *p* = 0.08). Furthermore, n-3 PUFA administration induced significantly increased proliferation in both sham (sham n-3 PUFA = 375.6 ± 40.6 BrdU^+^ cells) and immunotoxic lesioned groups (sap n-3 PUFA = 583.3 ± 68.7 BrdU^+^ cells; sham n-3 PUFA vs. sham oil, *p* = 0.001; sap n-3 PUFA vs. sap oil, *p* = 0.01; Fig. 6d, h). Then, the sub-populations of NSCs/neural progenitors which contribute to the increased proliferation observed in the n-3 PUFA treated groups were evaluated by using two specific cell markers: SOX2, which is expressed in the NSCs and in the early differentiating progenitors, and DCX, a specific cell marker of late differentiating and post-mitotic neuroblasts. A significant increase in SOX2^+^ cells was found in both n-3 PUFA groups (sham n-3 PUFA = 20,616 ± 384.2 SOX2^+^ cells; sap n-3 PUFA = 24,168 ± 553.4 SOX2^+^ cells) with respect to their oil-treated counterparts (sham oil = 16,251 ± 1229.2 SOX2^+^ cells; sap oil = 16,560 ± 427.53 SOX2^+^ cells; sham n-3 PUFA vs. sham oil, *p* = 0.01; sap n-3 PUFA vs. sap oil, *p* < 0.001; Fig. 6e, h), while the n-3 PUFA-dependent increase in DCX^+^ cells was significant only in the immunotoxic lesioned group (sap oil = 177.8 ± 39.3 DCX^+^ cells; sap n-3 PUFA = 408.6 ± 33 DCX^+^ cells; sap n-3 PUFA vs. sap oil, *p* < 0.007; Fig 6f).

#### Astrogliosis

To assess a putative anti-inflammatory role of n-3 PUFA treatment, the number of GFAP^+^ cells in the DG of the four experimental groups was measured. Cholinergic depletion induced a significant increase in GFAP^+^ cells (sham oil = 8552.7 ± 689 GFAP^+^ cells; sap oil = 11,476 ± 883.2 GFAP^+^ cells, sap oil vs. sham oil, *p* = 0.02), which was interestingly counteracted by the n-3 PUFA treatment (sap n-3 PUFA = 8312.7 ± 761.9 GFAP^+^ cells; sap n-3 PUFA vs. sap oil, *p* = 0.01; Fig. 6g).

#### Biochemical analyses

**Fig. 7:**
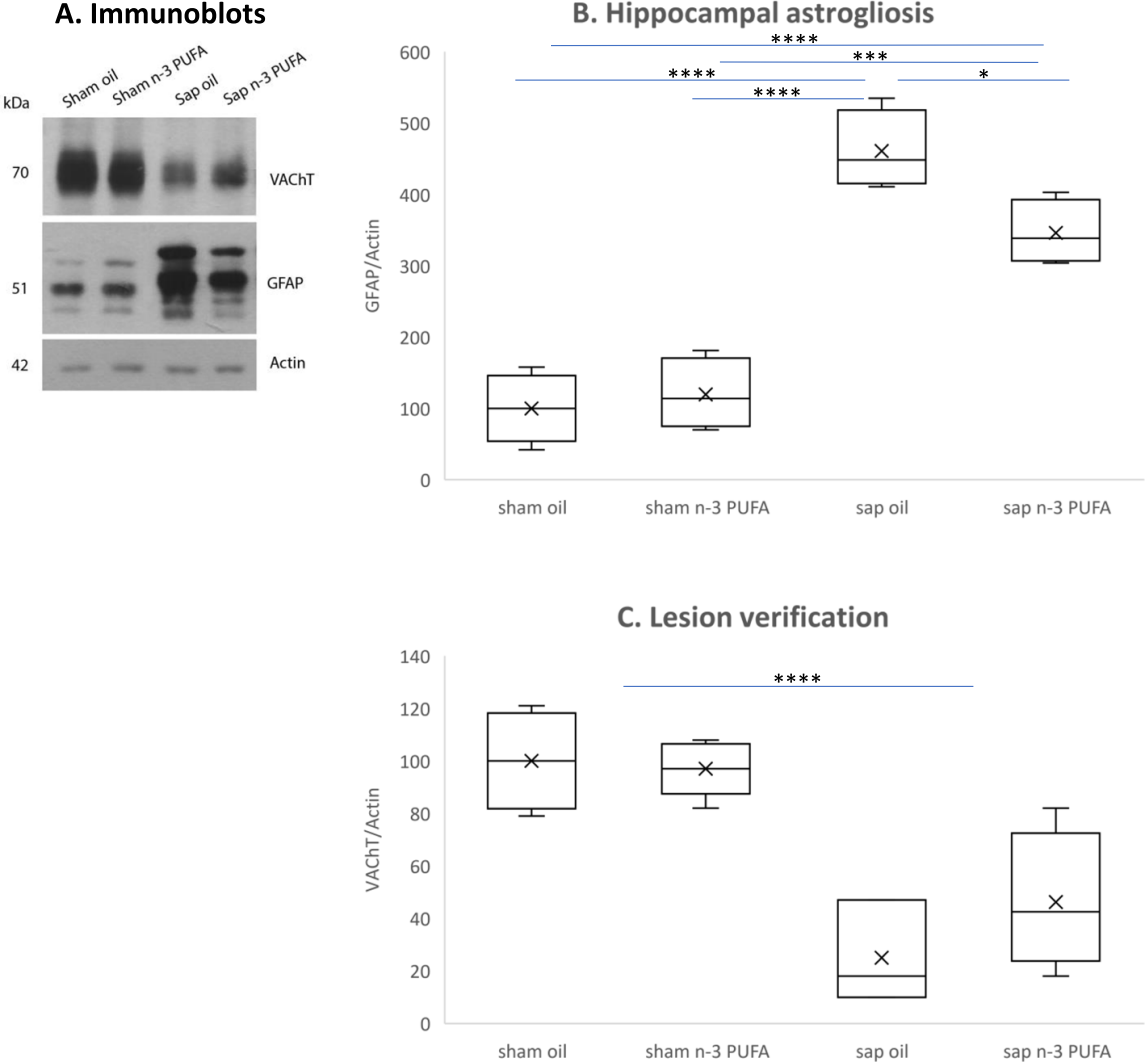
Hippocampal GFAP and VAChT immunoblotting results. **a** The figure shows representative immunoblot of total hippocampal proteins from the four experimental groups. **b**, **c** The histogram shows densitometric quantification of GFAP (**b**) or VAChT (**c**) changes in gray values, expressed as % of sham oil group. Data of the four experimental groups are depicted as mean and S.E., actin was used as loading control. (GFAP analysis: *n* = 4 per group; VAChT analysis: sham oil, n = 4; sham n-3 PUFA, *n* = 5; sap oil, *n* = 3; sap n-3 PUFA, *n* = 4). Statistical significance of the post-hoc comparisons between sap oil and the remaining groups: *p < 0.05; ***p < 0.001; ****p < 0.0001)

To examine whether the n-3 PUFA exert an anti-inflammatory activity in our mouse model, we analyzed the hippocampal GFAP levels (Fig. 7a, b). The two-way ANOVA showed significant lesion effects, and significant diet x lesion interaction (lesion: *F*_1,12_ = 139.4, *p* < 0.0001; diet: *F*_1,12_ = 3.65, *p* = 0.08; diet x lesion: *F*_1,12_ = 7.32, *p* = 0.02). In particular, post-hoc comparisons showed that saporin-lesioned mice treated with n-3 PUFA showed reduced levels of GFAP, compared to oil-treated animals, whereas n-3 PUFA had no effect on sham-lesioned mice (sham oil vs. sap oil and sap n-3 PUFA, *p* < 0.0001; sham n-3 PUFA vs. sap oil, *p* < 0.0001; sham n-3 PUFA vs. sap n-3 PUFA, *p* = 0.0002; sap oil vs. sap n-3 PUFA, *p* = 0.03).

Lesion verification through immunoblot analysis indicate an extensive reduction of VAChT in the hippocampus of both saporin-lesioned mice (lesion: *F*_1,12_ = 41.98, *p* < 0.0001; diet: *F*_1,12_ = 0.88, *p* = 0.37; lesion x diet: *F*_1,12_ = 1.56, *p* = 0.23; Fig. 7a, c).

Collectively, these findings prove that in the presence of a massive hippocampal cholinergic terminal loss, n-3 PUFA treatment was able to blunt hippocampal astrogliosis in aged mice.

### Lipid analysis

#### n-3 PUFA brain levels

**Fig. 8:**
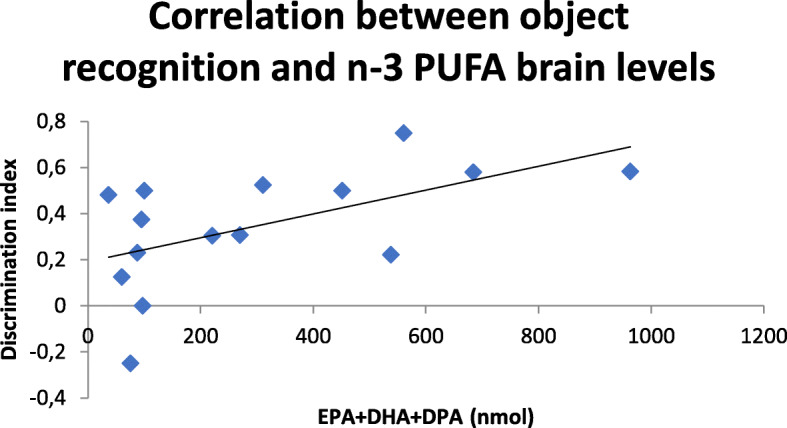
Correlation between object recognition and n-3 PUFA brain levels. Scatterplot representing the relationship between the discrimination index in NORT and total n-3 PUFA brain levels

The total amount of EPA+DHA+DPA in the brain of all experimental groups was measured (sham oil, *n* = 5; sham n-3 PUFA, *n* = 4; sap oil, *n* = 3; sap n-3 PUFA, n = 3; Suppl. Table 2). It tended to increase in n-3 PUFA-treated groups in comparison to oil-treated groups (*U* = 11, *p* = 0.049). No significant correlations of EPA+DHA+DPA levels with anxiety parameters (difference in time spent in closed vs. open arms in EPM: *r* = − 0.12, *p* = 0.68; number of buried marbles in MBT: *r* = − 0.06, *p* = 0.83) were evident. Interestingly, a significant positive correlation was found between EPA+DHA+DPA levels and the discrimination index in NORT (*r* = 0.56, *p* = 0.03; Fig. 8), suggesting an important role of n-3 PUFA brain concentrations as putative predictors of reduced cognitive deterioration during aging.

## Discussion

Nowadays, the necessity to increase our understanding of cognitive decline that characterizes the aging process and to develop new strategies for delaying and/or treating age-related neurodegenerative diseases, like AD, is becoming ever more urgent. AD is presently the most frequent, severe, and debilitating form of dementia worldwide [11, 108]. Its prevalence rises exponentially with age producing a huge burden on healthcare costs [24] and only palliative pharmacological treatments (inevitably accompanied by side effects) are now available [22–24].

Once the AD pathological cascade leading to neuronal death has started, delaying the neuronal loss is extremely difficult. The lack of resolutive treatments to halt AD makes thus the promotion of healthy lifestyles an alternative favorable approach to preserve neuronal populations in the aging brain for as long as possible. To this aim, one point essential for neuronal health and functionality is to reach an optimal supply of nutrients. Remarkably, over the past several years, mechanistic, epidemiologic, and interventional studies have demonstrated n-3 PUFA beneficial effects against brain aging and age-related cognitive decline [29, 30, 33, 41], with the most consistent effects against AD confined especially in the early or prodromal stages of the pathology [36, 48, 51, 52]. As an additional benefit, n-3 PUFA represent a low-cost, safe, and well-tolerated tool, without side effects for recommended doses [52].

In order to promote “nutrigeroprotective” interventions [109] for elderly affected by early AD, in the present study, we sought to further explore the neuroprotective potential of n-3 PUFA after the onset of a selective cholinergic depletion, which is retained to constitute one of the principal AD pathological events [53]. For this purpose, we examined whether the administration of n-3 PUFA after mu-p75-saporin lesions in aged mice could alleviate behavioral, morphological, and/or biochemical deficits.

Our results indicate an effective impact of n-3 PUFA in counteracting some functional and morphological deficits following cholinergic depletion and demonstrate increased regenerative patterns and decreased neuroinflammative components as possible underpinnings of the reduced cognitive decline and the concomitant reduced neuronal loss, even at old age.

Specifically, in sham-lesioned aged mice, the n-3 PUFA supplementation did not provoke any behavioral modification, apart from a reduced horizontal and vertical exploration during NORT that was present also in the saporin-lesioned mice treated with n-3 PUFA and could concur to a reduced stress response to novelty, as described elsewhere [110, 111]. Moreover, n-3 PUFA preserved hippocampal volumes and increased the proliferation of newborn neurons in the DG of sham-lesioned mice, in line with the few previous findings on aged mice [63, 64, 112, 113]. Astrogliosis levels were unaffected by n-3 PUFA treatment in sham-lesioned aged mice.

The cholinergic depletion per se induced anxiolytic effects and novelty recognition memory impairments in aged mice, without modifying social behaviors and passive coping responses. These behavioral results are in agreement with the reduction of anxiety [114, 115] and the presence of object recognition memory deficits [115–118] previously reported following cholinergic immunotoxic lesions in adult rodents. The anxiolytic pattern of our cholinergically depleted aged mice is also consistent with the increased exploration of open spaces in the EPM and reduced hiding in the MBT observed in mice with temporary and reversible inhibition of cholinergic neurons in the medial septum [67]. As for social interaction, it was scarce in our aged mice, as expected at late stage of life in rodents [65, 76, 119], and it was unaffected by the cholinergic lesion possibly due to a floor effect and/or to the sparing of striatal circuits linked to the rewarding properties of affiliative behaviors [54, 120, 121]. Regarding depressive-like responses, to our knowledge, this is the first study evaluating the performance of cholinergically depleted aged mice by using a two-session PT. We found no effect of cholinergic depletion on the increase in passive behavioral coping elicited by the re-exposure to the inescapable situation [122]. This finding is probably linked to a saporin lack of action on the dopaminergic accumbens/prefrontal networks which modulate stress coping and adaptation, in complementary fashion with mineralocorticoid and glucocorticoid receptors in the limbic brain [122, 123].

As for morphological and biochemical correlates, the cholinergic lesion increased hippocampal astrogliosis in agreement with similar findings in cholinergically depleted adult rats [107, 124] and aged mice [56], as well as in AD patients [125]. In the present study, we did not find any effect of the immunotoxic cholinergic lesion on hippocampal neurogenesis, in line with previous research in adult [126] and aged [56] mice. Hippocampal volumes were not significantly affected by the cholinergic lesion.

Remarkably, n-3 PUFA post-lesional supplementation regulated the anxiety alterations and reverted the novelty recognition memory impairment induced by the cholinergic depletion in aged mice. Similar beneficial effects of n-3 PUFA treatment on anxiety and novelty recognition memory have been demonstrated in other animal models during adulthood and aging [63, 72, 127–138].

The emotional and cognitive ameliorations here observed were complemented and supported by the increased n-3 PUFA brain levels and the modified hippocampal morphological features. Namely, in saporin-lesioned mice treated with n-3 PUFA, we detected preserved hippocampal volumes, increased proliferating cells and immature neurons in the DG, and diminished hippocampal astrogliosis with respect to control lesioned mice.

The n-3 PUFA efficacy in counteracting the age-related brain atrophy has been previously well-documented in rodent [28, 63, 64] and human [130–133] investigations. A preventive n-3 PUFA action against hippocampal neuronal loss has been already observed in aged mice when the n-3 PUFA administration preceded the cholinergic depletion [56]. Such an action may be attributed to n-3 PUFA positive influence in helping neurons to cope with aging by potentiating synaptogenesis and reducing apoptosis and glial degeneration [29, 56, 63].

In our study, the n-3 PUFA stimulation of neuronal progenitor proliferation (i.e., BrdU^+^ and SOX2^+^ cells) in the DG occurred after both sham and saporin lesions in aged mice, but the increase in neuronal differentiation of newborn neurons (i.e., DCX^+^ cells) persisted only after the immunotoxic lesion, probably to compensate for the cholinergic neuronal loss. Similar neurogenic effects of n-3 PUFA have been reported also in one of our previous studies [56], in which n-3 PUFA supplementation was administered in aged mice before cholinergic depletion. Notably, in case of traumatic brain injury [134, 135] and cerebral ischemia [136, 137], hippocampal neurogenesis is stimulated in order to recruit compensatory neural networks and to preserve the functions affected by the lesion. Further, in transgenic AD mice and in AD patients, hippocampal neurogenesis is increased either in response to the impaired neurotransmission and to disease-induced neuronal loss [138, 139].

Finally, although adult neurogenesis tends to decline with age [140], it is regulated by several extracellular cues, including hormones, growth factors, and neurotransmitters [141], such as acetylcholine [142–144]. Interestingly, n-3 PUFA enhancement of cholinergic transmission and neuronal membrane fluidity are both important modulators of neurogenesis [1, 3, 145, 146].

Anyhow, the neurogenic potential of n-3 PUFA may constitute the structural basis of the maintained mnesic performance of sap n-3 PUFA mice by virtue of the multiple implications of the hippocampal neurogenesis in mnesic retention [128, 147].

The n-3 PUFA-induced increase in neurogenesis as well as the hippocampal volume preserving occurred in the context of a decreased hippocampal astrogliosis. We focused on astrocytes since they constitute the most abundant type of neuroglial cells, have a key role in synaptic regulation (acting on formation, maturation, maintenance, pruning, and remodeling of synapses during aging and diseases) [148–151], and are well-characterized in aged humans [21, 152–154]. Aging may trigger the loss of normal function in astrocytes, which causes an intensified inflammatory state [155]. Moreover, AD brains are additionally characterized by a prominent reactive astrogliosis due to the progressive neurodegeneration [156]. In this study, while the hippocampal astrogliosis was detrimentally enhanced by the cholinergic depletion, the n-3 PUFA supplementation exerted a neuroprotective and anti-inflammatory role by lowering the increased astrogliosis hippocampal levels, in line with previous studies in aged subjects [29, 56, 63, 157]. However, we cannot exclude the involvement of microglia and oligodendroglia [158, 159] in modulating the n-3 PUFA effects on saporin lesions. In fact, there are strict functional neuroglial interactions; hence, after brain injury and in neurodegenerative disorders, the activated microglia may induce reactive astrocytosis which in turn drives death of neurons and oligodendrocytes [151].

Importantly, several dietary components (such as n-3 PUFA, antioxidants, and polyphenols) consumed in anti-inflammatory dietary patterns associated with slower rate of cognitive decline (such as Mediterranean diet) are reported to be able to inhibit neuroinflammation associated with AD via several immune pathways within the brain and indirectly from the gut microbiome and systemic circulation [160]. For example, fish-derived long chain n-3 PUFA may reduce the expression of pro-inflammatory cytokines in microglia and support resolution of inflammation in the brain [161, 162]. Moreover, in line with our findings, other studies on prodromal manifestations of AD, such as Aβ- or n-3 PUFA deficiency-induced depressive-like phenotypes, demonstrated that n-3 PUFA modulate neuroinflammatory activation pathways, especially in the hippocampal circuits [163, 164].

### Limitations

The first limitation of the present study may be the lack of young groups of mice lesioned and treated as the aged ones to also evaluate the age effect. For example, in the SIT, we found no significant effects of lesion or diet, but we can only hypothesize a floor effect linked to an age-related decrease in social interest based on previous studies in rodents [65, 76, 119]. It should be pointed out, however, that i.c.v. injections of the mu-p75-saporin in aged mice allow analyzing how the aging brain (and not the young one) reacts to the cholinergic depletion. Thus, since the majority of people with AD are older than 65 years, the use of aged animals does increase the translational power of the present study.

Secondly, we are aware that the use of aged mice exposes to the risk of an obvious reduction of the sample size, as the experiment goes by, due to increased mortality rate, especially following surgery. Therefore, we have a reduced number of measurements in some experimental groups, as for example in the n-3 PUFA brain levels and PT. Differently, the reduced number of subjects for SIT (in comparison to the other behavioral tasks) was due to video recording troubles, while the exclusion of some animals from NORT was due to the failure to meet the contact time criterion, maybe linked to the age-related reduced interest of aged animals also for inanimate stimuli [119]. Anyway, the overall sample size of the present study was calculated on the basis of power analysis and is comparable to our previous studies in which we performed similar behavioral, morphological, and biochemical analyses [56, 63].

A final potential limitation of the present study could have been that the animals received a prolonged n-3 PUFA supplementation via gavage. However, we feel to exclude any potential detrimental effect of it, since we previously demonstrated that 8 weeks of gavage did not modify the behavioral, morphological, and biochemical measurements in untreated naïve aged mice [63].

## Conclusions

The n-3 PUFA treatment represents a form of neuroplasticity promoter even at old age and in the presence of a massive cholinergic loss, thus supporting their use to delay AD pathology progression. Moreover, the discovery of a strict link between preserved hippocampal volume and neurogenesis and reduced astrogliosis as underpinnings of maintained cognitive and behavioral performances during aging shed new light on the n-3 PUFA mechanisms of action.

## Supplementary information

**Additional file 1: Suppl. Table 1.** NORT object preference, grooming and defecation data. Mean and S.E. of the following NORT parameters in the four experimental groups: preference for one of the two identical objects during the training trial (percentage of contact time with object A/total contact time with object A + object B); preference for the novel object during the test trial (percentage of contact time with the novel object/total contact time with the novel object + familiar object); grooming time (s) and number of defecations in all task trials.

**Additional file 2: Suppl. Table 2.** Brain levels of n-3 PUFA. Mean and S.E. of EPA + DHA + DPA brain levels in the four experimental groups.

## Data Availability

Analyses of all data produced during this study are included in the article (and its supplementary information files). Datasets will be available from the corresponding author on reasonable request.

## Abbreviations

AD: Alzheimer’s disease
ALA: Alpha-linolenic acid
Aβ: Amyloid-β
AP: Antero-posterior coordinate
BrdU: Bromodeoxyuridine
CA: Cornu Ammonis (Ammon’s Horn)
DAPI: 4′,6-Diamidino-2-phenylindole
DCX: Doublecortin
DG: Dentate gyrus
DHA: Docosahexaenoic acid
DPA: Docosapentaenoic acid
DV: Dorso-ventral coordinate
EPA: Eicosapentaenoic acid
EPM: Elevated plus maze
GFAP: Glial fibrillary acidic protein
i.c.v.: Intracerebroventricular
MBT: Marble burying test
MCI: Mild cognitive impairment
ML: Medio-lateral coordinate
n-3: PUFA omega-3 polyunsaturated fatty acids
NCI: Negative chemical ionization mode
NORT: Novel object recognition task
NSCs: Neural stem cells
p75NTR: p75 neurotrophin receptor
PBS: Phosphate buffer
PFA: Paraformaldehyde
PT: Porsolt test
Saporin: mu-p75-saporin
SIM: Selected ion monitoring
SIT: Social interactions test
SOX2: Sex-determining region Y-box 2
VAChT: Vesicular acetylcholine transporter
