# Supplementary material for: Behavioral, neuromorphological, and neurobiochemical effects induced by omega-3 fatty acids following basal forebrain cholinergic depletion in aged mice
Source: Alzheimers Res Ther. 2020 Nov 16;12:150. doi: 10.1186/s13195-020-00705-3 (PMC7667851; doi:10.1186/s13195-020-00705-3)
Supplement: Supplementary file 1 — Additional file 1: Suppl. Table 1. NORT object preference, grooming and defecation data. Mean and S.E. of the following NORT parameters in the four experimental groups: preference for one of the two identical objects during the training trial (percentage of contact time with object A/total contact time with object A + object B); preference for the novel object during the test trial (percentage of contact time with the novel object/total contact time with the novel object + familiar object); grooming time (s) and number of defecations in all task trials. [file 13195_2020_705_MOESM1_ESM.pdf]

Suppl. Table 1. NORT object preference, grooming and defecation data

Preference for object A		
	**Training trial**	
sham oil	47% ± 3%	
sham n-3 PUFA	51% ± 3%	
sap oil	48% ± 7%	
sap n-3 PUFA	51% ± 4%	

Preference for the novel object		
	**Test trial**	*Mann-Whitney U tests:*<br>- sap oil *vs.* sham oil: U=16.5, p=**0.013**;<br>- sap n-3 PUFA *vs.* sham n-3 PUFA: U=65, p=0.140;<br>- sham oil *vs.* sham n-3 PUFA: U=76.5, p=0.775;<br>- sap oil *vs.* sap n-3 PUFA: U=28.5, p=**0.049**;<br>- sap n-3 PUFA *vs.* sham oil: U=48, p=0.182.
sham oil	66% ± 5%	
sham n-3 PUFA	66% ± 5%	
sap oil	41% ± 7%	
sap n-3 PUFA	59% ± 5%	

Grooming (s)			
	**Habituation**	**Training trial**	**Test trial**
sham oil	11.09 ± 1.83	8.36 ± 2.96	9.55 ± 1.30
sham n-3 PUFA	9.93 ± 2.01	7.47 ± 2.26	11.60 ± 2.82
sap oil	6.78 ± 1.48	6.63 ± 1.21	12.11 ± 2.29
sap n-3 PUFA	9.62 ± 1.90	8.62 ± 1.85	7.92 ± 1.86

Defecations			
	**Habituation**	**Training trial**	**Test trial**
sham oil	0.64 ± 0.34	1.00 ± 0.19	0.18 ± 0.12
sham n-3 PUFA	1.13 ± 0.32	1.07 ± 0.25	1.27 ± 0.25
sap oil	0.67 ± 0.33	1.22 ± 0.32	1 ± 0.44
sap n-3 PUFA	1.31 ± 0.36	0.85 ± 0.22	1.46 ± 0.33

Suppl. Table 1. Mean and S.E. of the following NORT parameters in the four experimental groups: preference for one of the two identical objects during the training trial (percentage of contact time with object A/total contact time with object A + object B); preference for the novel object during the test trial (percentage of contact time with the novel object/total contact time with the novel object + familiar object); grooming time (s) and number of defecations in all task trials.
