# Supplementary material for: Behavioral, neuromorphological, and neurobiochemical effects induced by omega-3 fatty acids following basal forebrain cholinergic depletion in aged mice
Source: Alzheimers Res Ther. 2020 Nov 16;12:150. doi: 10.1186/s13195-020-00705-3 (PMC7667851; doi:10.1186/s13195-020-00705-3)
Supplement: Supplementary file 2 — Additional file 2: Suppl. Table 2. Brain levels of n-3 PUFA. Mean and S.E. of EPA + DHA + DPA brain levels in the four experimental groups. [file 13195_2020_705_MOESM2_ESM.pdf]

Suppl. Table 2. Brain levels of n-3 PUFA

EPA+DHA+DPA (nmol)	
sham oil	232.97 ± 75.10
sham n-3 PUFA	470.73 ± 127.67
sap oil	77.71 ± 10.75
sap n-3 PUFA	423.88 ± 272.43

Suppl. Table 2. Mean and S.E. of EPA+DHA+DPA brain levels in the four experimental groups.
